# High prevalence of spondyloarthritis and ankylosing spondylitis among familial Mediterranean fever patients and their first-degree relatives: further evidence for the connection

**DOI:** 10.1186/ar4154

**Published:** 2013-01-28

**Authors:** Servet Akar, Ozgul Soysal, Ali Balci, Dilek Solmaz, Vedat Gerdan, Fatos Onen, Mehmet Tunca, Nurullah Akkoc

**Affiliations:** 1 Department of Internal Medicine, Division of Rheumatology, Dokuz Eylul University School of Medicine, 35340 Izmir, Turkey; 2 Department of Radiodiagnostic, Dokuz Eylul University School of Medicine, 35340 Izmir, Turkey

## Abstract

**Introduction:**

Familial Mediterranean fever (FMF) is an auto-inflammatory disease characterized by recurrent attacks of fever and serositis. Limited data suggest that the prevalence of sacroiliitis is increased in patients with FMF. In our present study, we assessed the prevalence of spondyloarthritis (SpA), including ankylosing spondylitis (AS), among a cohort of FMF patients and their unaffected first-degree relatives (FDRs).

**Methods:**

The current study cohort comprised a consecutive group of 201 unrelated patients with FMF and 319 FDRs (≥ 16 years old). These subjects were examined according to a standard protocol.

**Results:**

A total of 157 FMF patients (78.1%) and 233 (73%) unaffected FDRs reported back pain. Fifteen FMF patients (7.5%) and nine unaffected FDRs fulfilled the modified New York (mNY) criteria for AS. One additional FDR with AS was identified after review of the medical records. None of the FMF patients with AS was HLA-B27 positive. The allele frequency of M694V among the FMF patients with radiographic sacroiliitis was significantly higher in comparison with those without sacroiliitis (OR 4.3). When compared with the general population, the risk ratios for SpA and AS among the FDRs of our FMF patients were 3.3 (95% CI; 2.0 to 5.5) and for AS 2.9 (95% CI; 1.3 to 6.4), respectively.

**Conclusions:**

Our study suggests that a) factors other than HLA-B27 play a role in the association of FMF and SpA/AS; b) *MEFV *gene variations may be one of the geographic/region-specific potential pathogenetic links between these two disorders in the Turkish population.

## Introduction

Familial Mediterranean fever (FMF) is an autoinflammatory disease characterized by recurrent febrile attacks of serositis [1]. Musculoskeletal features are common in patients with FMF [1,2], and arthritis is the second most common type of acute attack [1]. Typically this is acute self-limiting monoarthritis affecting the large joints of the lower limbs, and about 5% of FMF patients develop protracted arthritis, mostly in the hips or knees [2]. These features of the disease resemble spondyloarthritis (SpA). Moreover sacroiliitis, which is the hallmark of SpA, is reported to arise at a higher than expected frequency in both Turkish and Jewish FMF patients with musculoskeletal symptoms [3-5]. However the current data on the role of human leukocyte antigen (HLA)-B27 in the development of sacroiliitis in FMF patients are controversial [4,5].

The gene responsible for FMF (designated *MEFV*) is located on the short arm of chromosome 16 and encodes an immunoregulatory protein known as pyrin or marenostrin [6,7]. The M694V polymorphism within the *MEFV *gene is the leading variation among Turkish and Sephardic Jews and it has been demonstrated that arthritis is associated with the presence of M694V [1,2]. There is also some evidence that the M694V variation may be more frequent in FMF patients with sacroiliitis [4]. FMF is mainly prevalent in Jews, Turks, Armenians and Arabs, and the carrier frequency of *MEFV *variations in these populations has been reported to be as high as 39% [8,9]. This very high frequency in these populations has prompted interest in the *MEFV *status in seemingly healthy subjects. In this regard, both basal and peak acute phase protein concentrations have been found over several months to be greater in *MEFV *heterozygotes than in wild-type controls, regardless of the presence of gene variations [10]. It has been speculated that the upregulation of the inflammatory response may predispose *MEFV *carriers to certain types of inflammatory conditions. Indeed, febrile episodes, acute rheumatic fever and rheumatoid arthritis are reported to be higher in frequency in *MEFV *carriers than in healthy controls [11,12]. On the other hand, *MEFV *variations were tested previously in several inflammatory conditions and recent controlled studies have revealed that the M694V allele frequency is significantly increased in ankylosing spondylitis (AS) patients compared with controls [13-15].

To further elucidate these phenomena in our present study, we investigated the prevalence of SpA and AS in a cohort of FMF patients. We also evaluated the frequency of HLA-B27 and *MEFV *gene variations in patients with FMF and AS. To evaluate the role of *MEFV *in determining the susceptibility to SpA, we also examined the first-degree relatives (FDRs) of the patients in our study cohort who we expected to have a higher carriage rate of *MEFV *variations than the general population.

## Materials and methods

The first 258 FMF patients who had been invited to the outpatient clinic of our hospital to take part in an FMF survival study [16] were examined for possible inclusion in the subject cohort of our current study. Of these, 57 individuals were related to each other, thus, the first 201 unrelated patients were finally included in the analysis. All of these patients fulfilled the previously defined criteria of Livneh *et al. *for FMF [17]. The disease severity score for these FMF patients was calculated in accordance with the scoring criteria developed by the Sheba Medical Center [18]. FDRs (full siblings, parents and children) of our 201 FMF patients who were at least 16 years of age were also invited to the outpatient clinic for a formal evaluation.

All attending patients were examined using a standard protocol for capturing patients with SpA and AS. A face to face interview using a standardized questionnaire was undertaken in each case to obtain demographic and clinical data including the presence of back pain, the characteristics of inflammatory back pain (IBP), and other articular and extra-articular manifestations of SpA (peripheral arthritis, enthesitis, uveitis, dactylitis, psoriasis, inflammatory bowel disease, the presence of a preceding infection, and family history for SpA). The results of *MEFV *genotyping, the erythrocyte sedimentation rate (ESR) and C-reactive protein (CRP) levels were extracted from the patients' charts. All of the study participants completed the validated Turkish versions of the Bath ankylosing spondylitis disease activity index (BASDAI) [19,20] and the Bath ankylosing spondylitis functional index (BASFI) questionnaires [21,22]. In addition, all of the FMF patients in our cohort and their FDRs were clinically examined by a rheumatology fellow (OS) and a senior rheumatologist (SA).

The presence of IBP was judged according to the Calin [23] and Berlin [24] criteria. The diagnoses of SpA and AS were made based on the European Spondyloarthritis Study Group (ESSG) [25] and the modified New York (mNY) criteria [26], respectively. Axial SpA was defined according to the imaging arm of the Assessment of SpondyloArthritis International Society (ASAS) classification criteria [27].

Standard pelvic radiographs were obtained in all patients to assess the sacroiliac joints (SIJs). Each SIJ was scored on radiographs according to the mNY criteria [26] as follows: grade 0: normal; grade 1: suspicious; grade 2: minimal abnormality with small localized erosions, sclerosis without joint space alteration; grade 3: definite abnormality with erosion, sclerosis, and joint space widening or narrowing or partial ankylosis; grade 4: total ankylosis of joint.

In patients with IBP but normal pelvic radiographs, magnetic resonance imaging (MRI) of the SIJs was ordered. In FMF patients with IBP, assessment of HLA-B27 was also ordered. MRI was performed with a 1.5-Tesla (T) system (Philips Achieva, Eindhoven, Netherlands) using appropriate surface coils. Sequences were: (1) paracoronal T1-weighted (repetition time/echo time (TR/TE): 607 ms/18 ms; (2) paracoronal dual echo T2-weighted fat-saturated (spectral presaturation with inversion recovery (SPIR), TR/TE: 2874 ms/100 ms); (3) paracoronal dual echo proton density turbo spin echo (TR/TE: 2874 ms/12.5 ms). For all sequences slice thickness was 3 mm.

MRI was considered positive for active sacroiliitis according to the ASAS/Outcome Measures in Rheumatoid Arthritis Clinical Trials (OMERACT) MRI working group definition, that is the presence of at least one active lesion in at least two consecutive slices, or the presence of more than one lesion in only one slice [28].

After a series of training sessions for the standardization of assessment, all radiographs and all SPIR and T1-weighted sequences of paracoronal MR images were read and scored by two independent readers (SA and AB), who were blinded to the demographic and clinical features of the patients. Nineteen radiographs and twenty-four MRI scan series were re-scored by each reader for the calculation of intra-observer reliability. At the initial reading sessions there was moderate agreement between the two readers with regard to radiographic scoring of SIJs (the intraclass correlation coefficient (ICC) for the right and left SIJs were 0.647 and 0.682, respectively), and the two readers agreed on the presence of inflammation at any site of the SIJ on MRI in 91% of cases, with a к value of 0.623. ICC values for the first reader were 0.634 and 0.656, for the right and left SI joints, respectively, and the intra-observer reliability for diagnosing bone marrow edema was good (к = 0.750). ICC values for the second reader were 0.595 and 0.622 for the right and left SI joints, respectively, and к = 0.625 for MRI reading.

This study was approved by Dokuz Eylul University School of Medicine Ethical Committee for Clinical and Laboratory Research (protocol number 85/2009) and all patients gave written informed consent.

### Statistical analyses

Unless otherwise stated, the values in this paper are presented as the mean ± SD, or percentage as appropriate. Comparisons of categorical data between groups were made using the chi-square test. The Mann-Whitney *U*-test was used to analyze continuous data. To compare the observed frequency of SpA and AS in FDRs of our FMF patients, risk ratios were calculated using prevalence figures for the general Turkish population [29]. The intra- and inter-observer reliability for SIJ scoring according to the mNY criteria was analyzed by the calculation of the ICC (single measures for a single rater, average measures for different raters). Agreement for active inflammation identified on MRI was analyzed by cross-tabulation and к statistics. In cases on which two readers disagreed, a joint reading session was held in order to reach a consensus. All statistical tests were two-tailed and a *P*-value < 0.05 was considered statistically significant. Statistical analyses were performed using StatsDirect Statistical Software, version 2.0.0 (Cheshire, UK).

## Results

### Frequency of SpA and related features in FMF patients

The demographic features of the FMF study subjects are summarized in Table 1. Current or previous low back pain was reported by 157 (78%) of these patients, of whom 44 (28%) met the Calin criteria and 33 (21%) the Berlin criteria for IBP. None of our patients had uveitis and one had inflammatory bowel disease. Among our cohort, 70 cases (34.8%) fulfilled the ESSG criteria for SpA. Pelvic radiographs were obtained for 188 patients (93.5%) and radiographic sacroiliitis was diagnosed in 16 of these cases, of whom 15 (13 female (52%), median age (range) 46 (17 to 65) years) met the mNY criteria for AS (Table 2). The remaining patient with radiographic sacroiliitis was diagnosed with psoriatic arthritis.

**Table 1:** Demographic features of the familial Mediterranean fever patients and their first-degree relatives

Characteristics	Familial Mediterranean fever patients (FMF) (*n *= 201)	First degree relatives of FMF patients (*n *= 319)
Female, number (%)	105 (52%)	189 (59%)
Age, years, mean ± SD	39.6 ± 12.0	44.6 ± 15.5
Education level, mean ± SD	10.6 ± 3.7	8.6 ± 4.4
Disease severity score		
Mild, % individuals	37.5	-
Moderate, % individuals	13.5	-
Severe, % individuals	49.0	-
ESR, mm/h, mean ± SD	20.2 ± 17.7	17.5 ± 14.1
CRP, mg/L, mean ± SD	9.4 ± 19.7	4.2 ± 6.0
BASFI score, mean ± SD	0.9 ± 1.4	1.0 ± 1.6
BASDAI score, mean ± SD	2.4 ± 2.0	2.3 ± 2.1

ESR, erythrocyte sedimentation rate; CRP, C-reactive protein; BASDAI, Bath ankylosing spondylitis disease activity index; BASFI, Bath ankylosing spondylitis functional index.

**Table 2:** Characteristics of patients with both familial Mediterranean fever (FMF) and ankylosing spondylitis

Patient number	FMF/FDR	Clinical criteria of mNY	Arthritis	Enthesitis	Uveitis	MEFV typing	HLA-B27 typing
1	FMF	Yes	Yes	Yes	No	M694V/M680I	Negative
2	FMF	Yes	Yes	Yes	No	M694V/V726A	Negative
3	FMF	Yes	Yes	Yes	No	M694V/M694V	Negative
4	FMF	Yes	No	No	No	M680I/V726A	Negative
5	FMF	Yes	Yes	Yes	No	M694V/M694V	Negative
6	FMF	Yes	No	No	No	M694V/M680I	Negative
7	FMF	Yes	Yes	No	No	M694V/M694V	Negative
8	FMF	Yes	Yes	No	No	M694V/M694V	ND
9	FMF	Yes	No	Yes	No	M694V/M694V	ND
10	FMF	Yes	Yes	No	No	M694V/M694V	Negative
11	FMF	Yes	No	Yes	No	M694V/W	Negative
12	FMF	Yes	Yes	Yes	No	M694V/M694V	Negative
13	FMF	Yes	Yes	No	No	M694V/M694I	Negative
14	FMF	Yes	No	Yes	No	M694V/M694V	Negative
15	FMF	Yes	Yes	No	No	M694V/M694V	Negative
16	FDR	Yes	Yes	Yes	No	W/W	Negative
17	FDR	Yes	No	No	No	ND	Negative
18	FDR	Yes	No	No	No	ND	Negative
19	FDR	Yes	No	No	No	ND	ND
20	FDR	Yes	Yes	No	Yes	M694V/W	ND
21*	FDR	Yes	No	No	No	NA	NA
22	FDR	Yes	No	No	No	ND	ND
23	FDR	Yes	No	No	No	ND	ND
24	FDR	Yes	No	No	No	ND	ND
25	FDR	Yes	No	No	No	ND	ND

*Patient identified by medical record review. FDR, first-degree relative; M, male; F, female; ND, not done; NA, not available; mNY, modified New York.

The results of *MEFV *genotyping in our FMF patients with radiographic sacroiliitis are shown in Table 2. *MEFV *variant analysis was available for 146 FMF patients who did not have sacroiliitis and both the allele frequency (23/30 vs 126/292; *P *= 0.0005, odds ratio (OR) 4.3, 95% CI 1.8, 10.4) and the homozygosity rate (9/15 vs 30/146; *P *= 0.002, OR 5.8, 95% CI 1.9, 17.6) for M694V were found to be significantly increased in the FMF patients with AS. The carriage rate of the M694V polymorphism was found to be higher in patients with both FMF and AS but this finding was not statistically significant (OR 7.3, 95% CI 0.9, 57.1). HLA-B27 was genotyped in 13 patients with sacroiliitis and all were negative.

MRI analysis of the SIJs was obtained in 23 out of the 29 FMF patients in our cohort who had IBP, but not radiographic sacroiliitis. Four patients were found to have bone marrow edema on MRI, all of whom were carriers for M694V (one homozygous, one compound heterozygous (M694V/V726A), and two heterozygous) and negative for HLA-B27. In total 18 patients were classified with axial SpA according to the imaging arm of the ASAS criteria. Two patients with sacroiliitis on imaging did not fulfill the entry criterion of age at onset of symptoms as specified in the ASAS criteria. Hence, in our FMF patients the frequency of AS was 7.5% and the frequency of axial SpA was 8.9%.

### Frequency of SpA and related features in the FDRs of the FMF patients examined in this study

**Figure 1:**
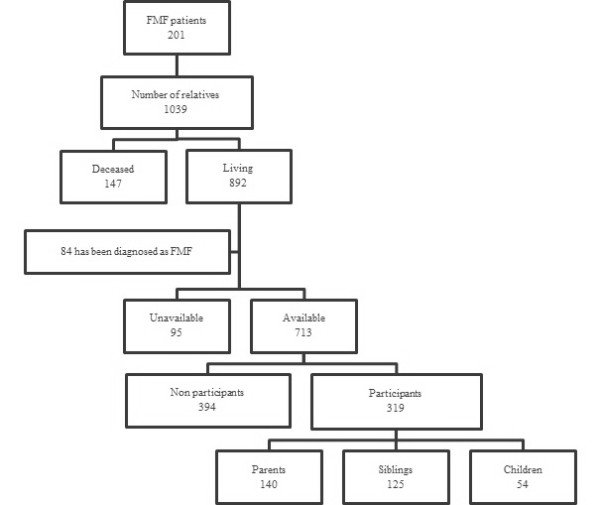
**Flow-chart summarizing the participant recruitment process used in this study**. FMF, familial Mediterranean fever.

In the second part of our study, we included the FDRs (16 years and over) of the 201 probands in our patient cohort. Of the total 1,039 FDRs of these patients, 892 were alive at the time of the study. We were unable to contact 95 of these FDRs after at least three attempts, and another 84 FDRs who had also been diagnosed with FMF were excluded from the study. Of the remaining 713 FDRs, 319 agreed to participate. The case recruitment process for FDRs of our FMF patients is summarized in Figure 1 and some of the demographic and clinical features of our study groups are shown in Table 1.

An episode of low back pain, even if in the distant past, was reported by 233 (73%) of the 319 FDR individuals. The Calin and Berlin criteria for IBP were met by 32 (13.7%) and 20 of these subjects (8.6%), respectively. A pelvic radiograph was taken in 277 (86.8%) FDRs of whom nine had unequivocal sacroiliitis as determined using the mNY criteria. On reviewing the medical records of the 394 FDRs who did not attend, one additional AS patient was identified. MRI analysis of the SIJs was performed in 19 out of the 27 FDRs of FMF patients who had IBP but not radiographic sacroiliitis. Bone marrow edema was detected by MRI in seven of these cases. Twenty-six FDRs fulfilled the ESSG criteria for SpA. Eleven (1.5%) FDRs were classified as axial SpA according to the ASAS criteria. Five patients with AS and one with sacroiliitis on MRI could not be diagnosed as axial SpA since they did not fulfill the entry criterion for age at onset, or duration of symptoms as per the ASAS criteria. Six FDRs had psoriasis, three had uveitis and one had inflammatory bowel disease. One FDR who had uveitis also had bilateral grade four sacroiliitis. Moreover, two individuals with Behcet's syndrome, two with rheumatoid arthritis and one with undifferentiated arthritis were identified among the FDRs. The prevalence of SpA (according to the ESSG criteria) and AS in the 713 FDRs was calculated as 3.6% and 1.4%, respectively. Based on a general population prevalence of 1.09% for SpA (according to the ESSG criteria) and of 0.49% for AS, as observed in a previous study conducted in our region [29], the risk ratio for SpA was estimated at 3.3 (95% CI 2.0, 5.5) and at 2.9 (95% CI 1.3, 6.4) for AS among the FDRs of FMF patients. When only the parents (*n *= 275) were included in this analysis, the corresponding risk ratios were 4.9 (95% CI 2.7, 9.0) and 3.7 (95% CI 1.3, 10.4), respectively.

## Discussion

The results of our present study reveal increased prevalence of SpA and AS among FMF patients. Our results thus confirm previous observations. The first case of FMF in association with AS was described in 1963 [30]. Thereafter, this association was reported in numerous other case reports. However, there are only a few studies to date that have systematically evaluated the relationship between FMF and SpA. In the first such study of this nature, 3,000 FMF patients were initially screened for the presence and manifestations of SpA [5], and among them, 160 patients with chronic arthritis were included in the subsequent study. In the aforementioned study, SpA was defined as the presence of chronic arthritis, inflammatory back/neck pain and sacroiliitis. Of the 160 FMF patients with chronic arthritis, 11 met the criteria for SpA of FMF (and actually all the subjects in this previous cohort fulfilled the New York criteria for AS). Moreover, the prevalence of SpA was estimated as 0.4% among the 3,000 initial patients. The HLA-B27 test was negative in all cases of SpA among these FMF patients. The authors excluded three other patients who had bilateral sacroiliitis, bamboo spine or were HLA-B27 positive. Although these cases met the authors' criteria of SpA of FMF they were interpreted as suffering from AS that coincided with their FMF, and were therefore clinically and genetically different from the other 11 patients. However, they also considered the possibility that these three patients might represent a more severe form in the clinical spectrum of FMF-related SpA, perhaps associated with the presence of HLA-B27.

In another earlier study from Turkey, the clinical and demographic features of 503 FMF patients were evaluated and the phenotypic differences between patients with and without amyloidosis were analyzed [3]. In this previous study, the prevalence of clinically and radiologically proven sacroiliitis was 6% (3/50) in patients with amyloidosis, and was 11% (50/453) in patients without amyloidosis. Overall, the frequency of sacroiliitis was 10.5%. However, the authors did not provide detailed definitions of clinical or radiographic sacroiliitis or information regarding the *MEFV *or HLA-B27 status of their FMF patients with sacroiliitis.

Recently in another study from Turkey [4], the authors retrospectively reviewed the medical records of 256 FMF patients to evaluate the presence of one or more musculoskeletal manifestations (inflammatory low back pain, arthritis and enthesitis). Of the 70 FMF patients with musculoskeletal findings 55 agreed to participate in the study. Direct radiographs of the SIJs were used to grade the sacroiliitis and MRI analysis of the SIJs was performed in patients with sacroiliitis grade 0, 1 or 2 as determined on direct radiography. In the abovementioned study there were eight patients (3.1%) diagnosed with a grade 3 to 4 sacroiliitis by direct radiography. All of these cases were male and were HLA-B27-positive, and none had signs of vertebral involvement. There were an additional 10 patients (3.1%) with sacroiliitis identified on MRI, all of whom were HLA-B27-negative. Overall the frequency of sacroiliitis among all FMF patients was reported to be 7%.

The main difference between our current study and previous reports such as those discussed here is that we evaluated all of the cases in a cohort of 201 patients rather than a select group of patients with articular or musculoskeletal involvement. In addition to the increased prevalence of SpA and AS found among our unselected FMF patients, we show from our current analysis that all of our tested patients with AS were negative for HLA-B27. This finding suggests that factors other than HLA-B27 play a role in the association of FMF and AS. *MEFV *itself may be the link between these two disorders since we also revealed an association between the development of AS in FMF patients and the M694V variation in the *MEFV *gene. This is in line with a previous observation from Turkey [4] which showed that M694V is the most common variation in FMF patients with both radiographic and MRI evidence of sacroiliitis. However, in the latter study most of the patients with radiographic sacroiliitis had only one *MEFV *variant and all of them were HLA-B27 positive. Moreover, in our previous report, we reviewed 22 adult case descriptions [31], of which *MEFV *gene variants were analyzed in 15 cases. Twelve patients were homozygous for M694V, two patients were compound heterozygotes (M694V/M680I; M680I/V726) and one patient had a single polymorphism (M680I/-).

To further delineate the role of *MEFV *in the susceptibility to SpA and AS, we also assessed the FDRs of FMF patients who were predicted to have a higher carrier frequency. We also analyzed the parents of our probands for obligate carrier status. We found significantly increased frequency of AS and SpA in the FDRs of FMF patients. In addition, the frequency of AS among the parents of our FMF patients was also high in comparison with the general population.

In FMF patients, mutations are found throughout the *MEFV *gene; however, those producing the most severe phenotype are clustered in exon 10, which encodes the B30.2/SPRY domain (PRYSPRY), at the C terminus of the pyrin protein. The exact function of pyrin still remains somewhat controversial. N-terminal pyrin appears to activate nuclear factor-κB (NF-κB) through the increased calpain mediated degradation of inhibitor of NF-κB (IκB)-α [32]. Recently, it was demonstrated that pyrin can interact with the apoptosis-associated speck-like protein (ASC), which has a caspase-recruitment domain (CARD) [33]. In addition to its role in apoptosis, ASC also nucleates inflammasome complexes through the homotypic interactions of its pyrin domain and CARD with NLRP proteins and inflammatory caspases, respectively [34]. Thus, ASC may mediate the activation of IL-1. The direct interaction of pyrin with ASC also uncovers potential molecular mechanisms for the abrupt onset inflammatory attacks associated with FMF [32].

Although the association between AS susceptibility and the class I molecule HLA-B27 is one of the strongest known HLA disease associations, the molecular mechanisms underlying disease pathogenesis still require clarification. In fact, the inability to explain this association on the basis of antigen presentation and major histocompatibility complex region, which reveal only half of the genetic susceptibility to AS, has led to alternative hypotheses [32]. The IL-1 pathway might be one of the pathogenetic mechanisms involved in AS. Candidate gene analyses have implicated the IL-1 cluster of genes as an AS susceptibility locus [35] and subsequent meta-analysis of whole genome linkage scans supported the linkage of chromosome 2q (IL-1 gene cluster) with AS [36]. Further, endoplasmic reticulum associated aminopeptidase 1 (*ERAP1*), a gene that was shown to be the strongest non-*MHC *gene associated with AS [37], also modulates the proinflammatory cytokines IL-1, IL-6, and TNF by cleaving their receptors at the cell surface [32].

In our present study, consecutive patients were included in order to minimize a possible bias for recruitment of patients with a history of SpA or AS, and a large number of relatives were also assessed. We evaluated both the patients and their FDRs using the same standardized protocol, which included a screening questionnaire, clinical examinations and radiography. One of the main limitations of our study may be its hospital-based nature. The frequency of more severe disease and allied conditions may be higher in those patients attending hospital visits regularly. Similarly, the frequency of symptomatic individuals may be higher in those relatives who agreed to participate in the study.

Unfortunately, we could not perform genetic analysis in the FDRs due to lack of resources, thus we could not use Amor, or the HLA-B27 arm of the ASAS classification criteria. Another limitation of this study was that only 319 (45%) of the available FDRs could be examined. However, our calculated prevalence rates represent the minimum prevalence, since we assumed that all of the potential subjects who did not participate in this study did not have SpA.

## Conclusions

The results of our present study not only confirmed an increased prevalence of SpA and AS among FMF patients, but also indicated for the first time that SpA and AS are more common among the FDRs of FMF patients than in the general population. The increased prevalence of SpA, including AS, among FMF patients as well as in their FDRs, provide additional evidence that there is a link between SpA/AS and FMF. The higher frequency of M694V among FMF patients with radiographic sacroiliitis than those without this disorder, suggest that *MEFV *gene variations may be the potential pathogenic link between the two disorders and can be recognized as a geographic region-specific risk factor for SpA/AS, affecting a common inflammatory pathway of IL-1 in the pathogenesis of these diseases.

## Abbreviations

AS: ankylosing spondylitis; ASAS: Assessment of SpondyloArthritis International Society; ASC: apoptosis-associated speck-like protein; BASDAI: Bath ankylosing spondylitis disease activity index; BASFI: Bath ankylosing spondylitis functional index; CARD: caspase-recruitment domain; CRP: C-reactive protein; ERAP-1: endoplasmic reticulum associated aminopeptidase 1; ESR: erythrocyte sedimentation rate; ESSG: European Spondyloarthritis Study Group; FDRs: first-degree relatives; FMF: familial Mediterranean fever; HLA-B27: human leukocyte antigen- B27; IBP: inflammatory back pain; ICC: intraclass correlation coefficient; IκB: inhibitor of nuclear factor-κB; IL: interleukin-1; *MEFV*: Mediterranean fever gene; MHC: major histocompatibility complex; mNY: modified New York; MRI: magnetic resonance imaging; NF-κB: nuclear factor-κB; OMERACT: Outcome Measures in Rheumatoid Arthritis Clinical Trials; OR: Odds Ratio; SIJ: sacroiliac joint; SpA: spondyloarthritis; SPIR: spectral presaturation with inversion recovery; TNF: tumor necrosis factor.

## Competing interests

The authors declare that they have no competing interests.

## Authors' contributions

SA, DS and NA participated in the design of the study, acquisition, analysis and interpretation of data and were involved in drafting the manuscript; OS participated in the design of the study, acquisition of data and was involved in drafting the manuscript; AB and FO participated in the design of the study, acquisition and interpretation of data and was involved in drafting the manuscript; VG participated in the design of the study, acquisition of data and were involved in drafting the manuscript; MT participated in the design of the study, acquisition of data and was involved in drafting the manuscript. All authors have given final approval of the manuscript to be published.
